# Supplementary material for: Resting-state EEG dynamics help explain differences in response control in ADHD: Insight into electrophysiological mechanisms and sex differences
Source: PLoS One. 2023 Oct 5;18(10):e0277382. doi: 10.1371/journal.pone.0277382 (PMC10553225; doi:10.1371/journal.pone.0277382)
Supplement: Attachment — Submitted filename: Response to Reviewers.docx [file pone.0277382.s002.docx]

**Response to reviewers**

**Reviewer #1:**

**Statistical p-values should be given with * referring p<0.05 and ** referring p<<0.05 in tables.**
Consistent *p*-value reporting was a point of concern for reviewer #2 as well. Tables have been modified as suggested, and we have also standardized our approach: *p*-values for both significant and non-significant effects referred to in writing are now reported, and all effects less than .001 have been written as *p* < .001. Moreover, since there are no *p* < .001 effects displayed in Figure 4, this value has been removed from the legend.

**The authors should discuss the findings in recent studies including graph theoretical connectivity analysis in ADHD, doi: 10.1016/j.bspc.2022.103626.**
This is an interesting article that we appeared to have overlooked. This article highlights the utility in measuring the modularity of EEG functional networks to distinguish ADHD from controls. As such, it has since been incorporated into the manuscript (section 1.1).

**How did the authors compare the groups ? what are the features (i.e. indicators) ?
Did they check the reliability of the results by examining several methods different from each other.**

The reviewer brings up a good point on sensitivity analyses (using several different methods to ensure results are not too closely dependent on a single approach). The purpose of testing various hidden Markov models, each with a different number of states (section 3.4) was to account for this. However, in light of these comments (and similar comments from reviewer #2), we feel as though the purpose of these analyses was not clearly specified.

To address this, we have since added a brief paragraph to the beginning of section 3.4 better explaining the purpose of these sensitivity analyses.

**Reviewer #2**

**An interesting study aiming to pinpoint the impact of dysregulated cortical network / response-control pathway in individuals with Attention Deficit Hyperactivity Disorder. The study benefits from a good sample size, using psychometrically validated scales, and the analyses appear thorough.**

We would like to thank the reviewers for recognizing the strengths of the paper.

**My biggest issue is that many readers may not be familiar with the Hidden Markov Modelling statistical approach. The way the results are presented and discussed rarely refer to EEG frequency bands that define each of the states the authors identified in their analyses. On the limited occasions that they do so (e.g. p22 where state 2 defined as relatively high alpha/beta and relatively low delta/theta), it enables the reader to contextualise the findings with regard to functional significance of EEG frequency bands in conjunction with ADHD. However, throughout the majority of the results and discussion it is unclear exactly what the results mean. A further example of this is in section 3.4 (p25) where the authors amend their analysis approach to explore states 3, 5, & 6 (previously it was 4 states). This needs to be clarified throughout; especially as the foreshadowing of the results (page 12) identified that it was the power of the cortical networks that explained the response control differences. This required detail will make the reading of this paper considerably more salient.**

The reviewer makes a good point. We have attempted to address this concern in multiple ways.

First, we have added a visualisation to Figure 2 showing the specific oscillatory characteristics (i.e., the model coefficients) of each state. We hope visually displaying this crucial piece of the results early on will help readers better contextualize the subsequent findings.

Second, to further reduce the cognitive load of readers, we have replaced mentions of 'state 2' in the discussion with the term 'high-alpha state', and ‘state 4’ with ‘hypo-activity state’. Rather than referring to an arbitrary number, these descriptions refer to the characteristics of the states themselves. We hope this will also help readers better contextualize our results.

Finally, we have added some writing to the introduction that clarifies the advantage of using hidden Markov models, in layman's terms. We believe this nicely compliments the more advanced discussion found in the methods.

In addition, we hope the section 3.4 paragraph added in response to reviewer #1 (clarifying the sensitivity analyses that were conducted) addresses the concerns that reviewer #2 had with us exploring 3, 5, and 6 state models.

**Other comments:**

**Intro:**

**p6/7 - there are no hypotheses for sex differences - do the authors have a priori direction for these?**

We debated the impact that sex might have on the relationship between resting state dynamics and response control while putting together our pre-registration. However, since the relationship between electrophysiology and response control was itself unclear (motivating the study), it was difficult to predict the impact that sex differences might have. Because of this, we opted to leave the directionality of the hypothesis unspecified.

**This also ties in with issues regarding structure of the introduction which deviates from standard formatting of a research article.**

We believe the reviewer is referring to the fact that we did not find support for our primary hypothesis (focused on modularity), but rather, a closely-related secondary hypothesis (focused on oscillator power). The decision to frame the introduction in this way was in the interest of transparency. We felt that reframing our introduction to be more in line with our results (rather than in line with our pre-registered report) would artificially inflate the strength of our results by suggesting that we found exactly what we set out to find (a form of HARKing: ‘hypothesizing after results are known’). Thus, we tried to strike a balance between maintaining the rationale from our pre-registration and incorporating relevant oscillatory-power literature such that our results did not come as a surprise to readers.

**Method:**

**p8 – the grouping and matching (lines 1-4) are unclear, as it reads that females were matched by age, and males were matched by subtype (not specified). Please reword accordingly.**

We agree that the writing in this section was unclear. We have since added a couple of sentences that more precisely characterize the procedure we used to select participants.

**p9 Section 2.3: CBCL – As it has been stated only 3 subscales were included, please clarify number of items used in the study and provide the Cronbach’s alpha. C3-SR-S / SCARED– please provide the Cronbach’s alpha values observed here.**

Cronbach’s Alpha for each scale, and the number of items within each CBCL subtest, have since been reported. We believe these highlight the high internal consistency of the scales.

**Please state which programme was used to analyse the data.**

We have since added two sentences (sections 2.4.2 and 2.4.3) that specify the versions of *R* and *MATLAB* used to conduct our analyses.

**The behavioural results in sections 3.1 and 3.2 are missing the inferential statistics, and have p values only reported, whereas inferential statistics have been included in section 3.2.2. Please be consistent throughout the results; another example is p15 ‘As shown in Table 2…’ three non-significant findings are referred to in text, but only one p value reported. Please also consistently state the direction of the result.**

These corrections have been made: inferential statistics have since been added, and the direction of effects have been stated.

**There are inconsistencies in statistical reporting also. It would be clearer to the reader to adhere to one format (p values) when referring to significance / non-significance rather than the variation presented in Tables 2 and 4. For example, Table 4 also includes a significant p value (state 3 power). If you have a reason to use this alternative format for significance reporting then please can you explain why. There are also inconsistencies in the body of the text in how the p value is reported for significant findings when the p value is less than .001. In some cases, p<.001 has be used, other times an exact p value is reported e.g. p = .0009 (p23).**

This has been corrected: *p-*values for all effects less than .001 are now described as “*p* < .001”. The tables have also been adjusted such that they use consistent *p*-value formatting.

**The table legends do not follow standard formatting, where the legend should be above the table. Similarly, the legends for the figures should be below the figure. Much text below the figures should actually be incorporated in the body of the text. On some occasions it has been included in text also, therefore there is repetition.**

We have corrected the repeated presentation of results in both the figure captions and body of the text by removing all redundant information from the captions (changes are particularly noticeable for the scatter plots). These captions are now considerably more succinct, in line with PLoS One guidelines.

**p18: Where several non-significant findings are combined in one statement, provide minimum p value i.e. p≥ as opposed to p>.**

This has been addressed.

**p20: HMM-Power dwell-times: age negatively correlated with dwell time in state 3 but coefficient is positive. Text and Figure 3 do not correspond.**

We apologize for the error included in this section. As can be seen in Figure 4, the coefficient should be negative. We have also double-checked to ensure there were no more oversights that led to an inconsistent reporting of results.

**The legend requires rewording as age is not a phenotype.**

The legend and caption for Figure 2 have been changed accordingly.

**p22: Figure 4 correlations – remove *** p<.001 as there are no results at this level of significance presented**

This part of the legend has been removed.

**p24: Figure 6 – text states increased dwell-times with increased state 2 – but coefficient is a negative correlation. Reword the females result to state non-significant rather than attenuated.**

The figure caption has been corrected.

**p25 – ‘in the four groups created by our two variables of interest’ - it is not clear what this means. Please check thoroughly throughout to ensure correct statistical reporting of all the results. This section would benefit from a summary of the key findings.**

We agree that this section was unclear, and have since described the exact procedure more clearly. We also added a short paragraph to the end of this section that summarizes the main findings.

**Discussion:**

**p28 – remove the inferential stats. Again, it is not clear what states 2 and 4 relate to therefore not clear how this concurs with Cai et al. (2018).**

These inferential statistics have been removed, and the claim that these findings relate to those from Cai et al. (2018) has been lessened.

**Please also see my general comments regarding the lack of reference to EEG frequency bands associated with each state. It is hard for the lay reader to follow how the findings fit with established literature.**

References to specific state numbers (i.e., “time in state 2”) have been replaced with descriptions that better capture the properties of the state (“time in the high-alpha state”).

**Minor comments:**

**Acronyms need to be written in full initially e.g. p4 line 4 states ‘dysregulated FPN – SAL interactions’, but the acronym for FPN is not stated till p33, I cannot find a full reference for SAL in the manuscript.**

Corrected.

**p5 reference Stam et a – missing the ‘l’**

Corrected.

**Method:**

**p7: Research ethics board – xxxx – is this a typo? It is not a blinded submission**

Corrected.

**Inconsistencies in reporting the ages of participants. The methods state ages 8-18 were targeted, the abstract states 8-16, the text in the results refers to 16.83. Once the demographic information in the results has been moved to the Method please be consistent regarding the age range.**

This inconsistency arose from the fact that our participant selection criteria allowed for participants aged 8 to 18, while the oldest participant that entered analysis was 16.83 years old. We have re-written part of section 2.1 so that this is clear.

**p8 lines 5-12 - signpost reader to section 2.3 regarding these materials.**

Corrected.

**p11 - below modularity equation. ‘Where □…’ – typo**

Corrected.

**Section 2.3: Connors – missing closing bracket (positive and negative impression**

Corrected.
